# Novel peptides with calcium‐binding capacity from antler bone hydrolysate, its bioactivity on MC3T3‐E1 cells, and the possible chelating mode

**DOI:** 10.1002/fsn3.4441

**Published:** 2024-09-20

**Authors:** Zhaoguo Wang, Xiaorui Zhai, Xingyu Xiao, Peijun Xia, Xi Chen, Yi Li, Linlin Hao, Yining Zhang

**Affiliations:** ^1^ College of Animal Science Jilin University Changchun China; ^2^ Department of Pediatric Endocrinology, Genetics and Metabolism, Children's Medical Center The First Hospital of Jilin University Changchun China

**Keywords:** antler bone, calcium chelating peptide, chelating mode

## Abstract

In this study, peptide‐calcium chelate was screened from antler bone hydrolysate, and its bioactivity on MC3T3‐E1 cells and its chelating mechanism were investigated. In vitro experiments showed that peptide‐calcium chelate promoted the differentiation and mineralization of MC3T3‐E1 cells. Subsequently, three novel calcium‐chelating peptides were obtained from antler bone hydrolysate using hydroxyapatite chromatography (HAC), Sephadex G‐25 gel filtration chromatography, and reversed‐phase high‐performance liquid chromatography (RP‐HPLC). Meanwhile, this work determined peptides' amino acid sequences as TKLGTQLQL, LETVILGLLKT, and KMVFLMDLLK based on LC–MS/MS. Then the present work prepared the three peptides, with the corresponding calcium‐chelating rates being verified as 87.68 ± 2.86%, 80.72 ± 0.93%, and 67.96 ± 0.98%, respectively. Fourier transform infrared (FTIR) spectroscopy, ultraviolet–visible absorption (UV–vis) spectroscopy, X‐ray diffraction (XRD), circular dichroism (CD), zeta potential, and molecular dynamics (MD) simulations were adopted to investigate the chelating mode of peptides with calcium ions. As a result, oxygen in the carboxyl group and the nitrogen in the amino group were related to calcium binding. In addition, the chelation site preferred the negatively charged carboxylate groups of Leu or Thr. The present work revealed that antler bone might be the new calcium‐chelating peptide source and elucidated their positive role in osteogenesis.

## Graphical abstract

This study identified that antler bone peptide‐calcium chelate promotes MC3T3‐E1 cell differentiation and mineralization. Two novel calcium‐chelating peptides were identified from antler bone hydrolysate with high chelation rates. Oxygen and nitrogen in the peptides' carboxyl and amino groups are involved in calcium binding, with a preference for negatively charged carboxylate groups of Leu or Thr.

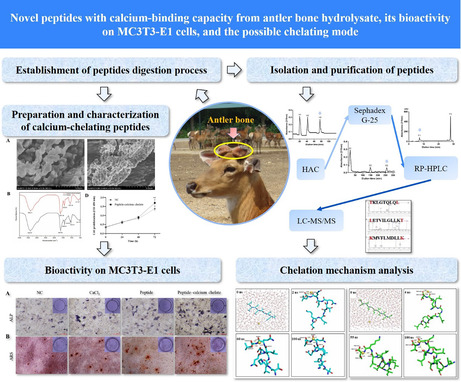

## 1 INTRODUCTION

Jilin province of China is a major deer breeding area in the world, and the current estimate annual production of antler is 307 tons (Wu et al., 2013). The antler, as the main product of deer, is a typical traditional medicine recognized by pharmacopeias of China, Korea, and Japan (Wang et al., 2019). Additionally, it is commonly used as a functional food and nutritional supplement in New Zealand, Canada, and the United States. The antler is the only completely regenerable organ found in mammals, with a rapid growth rate of up to 1.7 cm/day (Sui et al., 2014). The antler bone, located at the basal section of antler, has a low market value and is even not to be exported due to severe ossification (Li et al., 2014), whereas antler bone is rich in amino acid (402.97 ± 49.52 mg/g) and calcium (12.71 ± 1.07%) (Wu et al., 2013). It has previously been reported that the levels of proline, glycine, alanine, and arginine in the antler bone were higher than the tips of the antler, and these amino acids have been shown to bind to calcium ions through the R group (Tseng et al., 2014). Overall, antler bone is an abundant source of amino acids and a natural reservoir of calcium‐chelating peptides.

Calcium accounts for one of the essential nutrients within the human body, with prominent functions in bone health, muscle contraction, and nerve conduction (Tseng et al., 2014). Calcium insufficiency is associated with an increased risk of kidney stones and colon cancer (Reid & Bolland, 2019). The only source of calcium is from the diet (Hodges et al., 2019). However, it is difficult to obtain sufficient calcium from diet directly due to the formation of insoluble calcium salts in the intestine (Hodges et al., 2019). Inadequate calcium intake is a common occurrence in all countries and across all age groups (Zhu & Prince, 2012). Research on calcium supplements that can be absorbed effectively has been the focus of attention. Calcium‐chelating peptide, a recently developed calcium supplement, can form soluble peptide‐calcium complexes under certain conditions (Peng et al., 2017; Wu, He, et al., 2019). Peptide‐calcium chelate enhances calcium ion stability within the gastrointestinal environment, while further increasing calcium absorption because there are diverse amino acid residues that have abundant electron and ionic side chains, together with appropriate conformations (Wu, Cai, et al., 2019; Wu, Yang, et al., 2020). Therefore, we investigated the osteogenic bioactivity of peptide‐calcium chelate based on previous studies by cellular experiments on MC3T3‐E1 cells.

In recent studies, peptide‐calcium chelate biological function on MC3T3‐E1 cells was determined, and calcium‐chelating peptides were separated and purified in different sources and the binding mechanism was investigated by characterization methods (Liao et al., 2020; Malison et al., 2021; Peng et al., 2017; Walters et al., 2018; Wu et al., 2019a, 2019b). The present work focused on isolating calcium‐chelating peptides in antler bone hydrolysates by HAC, Sephadex G‐25 gel filtration chromatography, and RP‐HPLC. Structural characterization methods and molecular dynamics simulation were applied in detail to discover the possible chelating mechanism between the peptides and calcium ions. This study has important implications for the use of antler bone hydrolysate as calcium‐chelating peptide components in functional foods. The research also provides a theoretical basis for improving the utilization value of antler bone.

## 2 MATERIALS AND METHODS

### 2.1 Materials

This work obtained freshly prepared antler bone from CHANG SHENG Deer Industry Co., Ltd. (Jilin, China) and preserved it under −20°C for subsequent analysis. Neutrase (≥100 U/mg), papain (≥800 U/mg), alcalde (≥200 U/mg), trypsin (from porcine pancreas, ≥250 U/mg), and flavourzyme (≥20 U/mg) were provided by Yuanye Biotechnology Co., Ltd. (Shanghai, China). The remaining chemicals utilized in the present work were analytically pure and obtained.

### 2.2 Preparation of antler bone hydrolysate

The antler bone was sliced with a bone slicer, and slices were rinsed using deionized water (diH_2_O) until the pH became neutral, followed by drying at room temperature and smashing into powders. Later, this work added diH_2_O into powders at the 10:1 ratio (w/v), followed by 5 h (Yin et al., 2024) hydrolysis using alcalase (45°C, pH 9.5), neutrase (50°C, pH 7.5), trypsin (50°C, pH 8.0), papain (55°C, pH 6.0), flavourzyme (50°C, pH 7.5) at 3000 U/g powders (Wu, He, et al., 2019), separately. The mixtures were then heated for a 5 min period under 100°C for enzyme inactivation and hydrolysis termination, followed by 10‐min centrifugation at 10733 ***g*** and 4°C. Thereafter, this work harvested supernatants to analyze the calcium chelating rate and the degree of hydrolysis. After lyophilization, we preserved supernatants under −20°C for further analysis.

Next, to optimize the enzymatic conditions of the antler bone, the single‐factor test was performed. This work dissolved lyophilized hydrolysate into diH_2_O of different contents (2%–18%), and then enzyme was added with different dosages (1000–9000 U/g), followed by 1–9 h mixture incubation within the water bath under diverse pH (5.5–9.5) and temperature (30–70°C) conditions. Those optimal enzymatic conditions of the antler bone were determined according to the result of the calcium‐chelating rate and the degree of hydrolysis.

Based on the single‐factor experiment, three parameters, enzyme concentration, enzyme time, and concentration of substrate, were selected to design a three‐level three‐factor Box–Behnken Design (BBD) using Design Export software (Stat‐Ease, USA) (Zheng et al., 2018). The full experimental design with respect to experimental values for the responses is presented in Table 1. The model proposed for the response was:

$$\gamma =\beta_{0}+\sum_{i=1}^{3}\;\beta_{i}X_{i}+\sum_{i=1}^{3}\;\beta_{\mathrm{ii}}X_{i}^{2}+\sum \sum_{i<j=1}^{3}\;\beta_{\mathrm{ij}}X_{i}X_{j}$$

where *γ* is the dependent variable, while *X*_*i*_ and *X*_*j*_ are the independent variables. *β*_0_ is constant, and *β*_*i*_, *β*_*ii*_, and *β*_*ij*_ are the regression coefficient for linear, quadratic, and interaction terms, respectively.

**TABLE 1:** Response surface experimental design and results.

Run	(A) Enzyme concentration (U/g)	(B) Hydrolysis time (h)	(C) Concentration of substrate (%)	(D) calcium chelation capacity (%)
1	5000	7	6	27.59
2	5000	3	6	32.65
3	3000	3	10	29.28
4	7000	5	14	28.21
5	3000	5	6	33.05
6	5000	5	10	34.87
7	5000	3	14	30.78
8	5000	5	10	36.45
9	7000	5	6	31.97
10	5000	5	10	30.07
11	5000	5	10	34.89
12	7000	3	10	30.9
13	3000	5	14	36.71
14	7000	7	10	35.21
15	5000	5	10	27.34
16	3000	7	10	33.56
17	5000	7	14	32.79

### 2.3 Measurement of the degree of hydrolysis

This work measured hydrolysis extent through the ratio of α‐amino nitrogen (AN) to total protein nitrogen (TPN); of them, AN was measured by the formaldehyde titration approach. In brief, this work titrated 5 mL of enzymatic hydrolysate using 0.05 M NaOH until the pH became 8.2, followed by the addition of 10 mL of neutral formaldehyde as well as titration using 0.05 M NaOH until the pH value became 9.2. This work recorded the NaOH (0.05 M) volume utilized within the blank experiment (sample was replaced by diH_2_O) as *V*_0_, whereas that utilized following formaldehyde addition as *V*_1_. Thus, the present study determined AN as follows:

$$\text{AN}\;\left(g/\mathrm{mL}\right)=\left(\;V_{1}–\;V_{0}\right)\times 0.05\times 0.014/5$$

where 0.014 stands for nitrogen mass (g) equal to 1 mL of the 1 M NaOH, whereas 5 stands for sample volume (mL) during the titration process.

DH=AN×V/TPN×100

in which *V* stands for total enzymatic hydrolysate volume (mL), and TPN could be measured through Kjeldahl determination (Armas et al., 2006).

### 2.4 Peptide‐calcium chelate preparation

This work made the peptide‐calcium chelate through stirring at 50°C for 60 min, in which the concentration ratio of peptide to Ca^2+^ was 5:1 with the pH maintaining at 7.5, as previously described. Later, the mixed solution was blended with absolute ethanol at a volume of 1:9, followed by 10‐min centrifugation at 10733 ***g*** and 4°C. Afterwards, we named the precipitate peptide‐calcium chelate and lyophilized it for further analysis.

### 2.5 Calcium binding capacity analysis

This work measured calcium binding capacity by Wu et al.'s method (Wang et al., 2018; Wu, He, et al., 2019) after slight modification. After lyophilization, this work dissolved hydrolysate into the 5 mg/mL diH_2_O and mixed with CaCl_2_ solution (equivalent to Ca^2+^ 1 mg/mL) in equal volume. Then the mixture was subject to 60‐min stirring under 50°C, and the mixture pH was maintained at 7.5. Thereafter, the solution was blended with absolute ethanol (at a volumetric ratio of 1:9) under 4°C. The flame atomic absorption spectrophotometer was employed to determine total calcium content within the solution as well as calcium concentration within the supernatant following 10 min centrifugation at 10733 ***g*** and 4°C.

$$\text{Calcium chelation rate}=\frac{\text{total calcium in solution}-\text{calcium amount in supernatant}}{\text{total calcium in solution}}\times 100\%$$

### 2.6 Scanning electron microscope (SEM)

Specimens were applied onto an aluminum plate, followed by the deposition of a gold sputter coating. Subsequent imaging was carried out using the SU8010 scanning electron microscope (SEM) instrument manufactured by Hitachi, Japan. Imaging was conducted at an operational voltage of 5 kV.

### 2.7 Fourier transform infrared spectroscopy (FTIR) measurement

For exploring those possible binding sites between peptide and calcium, this work grinded 1 mg peptide or peptide‐calcium chelate sample uniformly using 100 mg of dry KBr, followed by compression in the transparent piece before subsequent use. Thereafter, this work obtained absorption spectra within the range of 400–4000 cm^−1^ by FTIR instrument (Thermo Nicolet iS5, USA) and employed Omnic software (Thermo Nicolet Co., Madison, WI, USA) for analyzing peak signals within those obtained spectra.

### 2.8 Cell proliferation assay

MC3T3‐E1 cells (purchased from American Type Culture Collection, ATCC) were maintained in α‐minimal essential medium (α‐MEM) supplemented with 10% fetal bovine serum (FBS) and 1% penicillin/streptomycin (P/S). The cells were incubated at 37°C in a humidified atmosphere composed of 95% air and 5% CO_2_. MC3T3‐E1 cells were seeded in 96‐well plates at a density of 1.2 × 10^4^ cells per well. Once the cell confluence reached 80%, the cells underwent various treatments, including vehicle CaCl_2_ (9.24 μg mL^−1^), peptides (46.2 μg mL^−1^), a mixture of CaCl_2_ (9.24 μg mL^−1^) and peptides (46.2 μg mL^−1^), and Pacific cod bone collagen peptide (BCP, 46.2 μg mL^−1^) (Peng et al., 2017). Cell proliferation was detected after a treatment time of 72 h. In addition, the effects of peptide‐calcium chelate on the proliferation rate of MC3T3‐E1 cells were examined at 0, 24, 48, and 72 h. Cell proliferation was assessed utilizing the CCK‐8 (Apexbio, United States) assay by measuring the absorbance of the cells at 450 nm using a microplate reader (TECAN, China). The obtained results were normalized and presented as relative ratios compared to the control. The cell proliferation rate was calculated as:

$$\text{Cell proliferation rate}=\frac{\left(\mathrm{As}-\mathrm{Ab}\right)}{\left(\mathrm{Ac}-\mathrm{Ab}\right)}\times 100\%$$

where As is the sample absorbance, Ab is the blank absorbance, and Ac is the control absorbance.

### 2.9 Osteogenic induction and alkaline phosphatase activity (ALP) assay

MC3T3‐E1 cells were inoculated at a density of 2.0 × 10^4^ cells/well in 24‐well plates and cultured in osteogenic differentiation media. The cells were treated with vehicle, CaCl_2_ (9.24 μg mL^−1^), peptides (46.2 μg mL^−1^), or a mixture of CaCl_2_ (9.24 μg mL^−1^) and peptides (46.2 μg mL^−1^) in osteogenic differentiation media (10 mM β‐glycerophosphate, 50 μg mL^−1^ ascorbic acid, 10 nmol dexamethasone) for 7 days. The media was replaced every 3 days, and differentiation was induced for a period of 21 days. After 7 days of osteogenic induction, the culture media was discarded, the cells were washed twice with PBS, and then the cells were lysed with passive lysis buffer for 30 min on ice. Then, the lysate was tested with the ALP activity assay kit (Nanjing Jiancheng, China) and incubated at 37°C for 15 min, and the OD value was measured at 520 nm using a microplate reader. The concentration of sample protein was determined using a BCA protein assay kit (Beyotime, China). The ALP activity was calculated as:

$$\mathrm{ALP}\;\text{activity}=\frac{\left(\mathrm{As}-\mathrm{Ab}\right)}{\left(\mathrm{Ac}-\mathrm{Ab}\right)}\times \frac{\text{concentration of phenol standard solution}}{\text{concentration of sample protein}}$$

where As is the sample absorbance, Ab is the blank absorbance, and Ac is standard absorbance. Total protein content was used to normalize ALP activity, and all assays were repeated at least three times. Cultured in osteogenic differentiation media for 7 days and stained for ALP using the BCIP/NBT ALP Color Development Kit (Beyotime, China) as described by Lijun Wang. (Wang et al., 2022).

### 2.10 Mineralization assay

MC3T3‐E1 cells were seeded at a density of 1 × 10^5^ cells/well in 12‐well plates. The cells were treated with vehicle, CaCl_2_ (9.24 μg mL^−1^), peptides (46.2 μg mL^−1^), or a mixture of CaCl_2_ (9.24 μg mL^−1^) and peptides (46.2 μg mL^−1^) in differentiation media for 28 days. The media was replaced every 2–3 days. After treatment, the cells were fixed in 4% polyoxymethylene for 30 min, stained with 1% Alizarin red S solution for 10 min, and washed 3 times with double distilled water (ddH_2_O). The stained deposits of calcium nodules were obtained using a camera (Leica, Germany). In order to quantify the degree of alizarin red staining area, the stained nodules were dissolved with 10% cetylpyridinium chloride for 30 min to quantify the degree of staining, and then the absorbance at 562 nm was measured using a microplate reader (Wang et al., 2020).

### 2.11 Isolation and purification of peptides from antler bone hydrolysate

#### 2.11.1 Hydroxyapatite chromatography (HAC)

This work dissolved lyophilized hydrolysate, which was prepared through a single‐factor test, into deionized water (diH_2_O) at 200 mg/mL, and then passed it through the 0.45‐μm filter membrane, followed by loading of solution onto a HAC column (Bio‐rad, Type I, 80 μm particle, USA) equilibrated with 5 mM phosphate buffer (pH 6.8). Later, we eluted the solution with PBS (pH 6.8) at the doses of 100, 200, and 400 mM at the 2.0 mL/min flow rate. Thereafter, elution monitoring at 215 nm was conducted. Each peak was pooled, followed by lyophilization to test the calcium chelating ability.

#### 2.11.2 Gel filtration chromatography (Sephadex G‐25)

Then this work dissolved the fraction showing the greatest calcium chelating capacity into diH_2_O at 100 mg/mL, followed by purification with Sephadex G‐25 column (GE Healthcare Co., Sweden), which was eluted by diH_2_O at the 0.5 mL/min flow rate. Later, this work measured the eluate absorbance (OD) value at 215 nm. Next, the fractions that showed the greatest calcium‐binding capacity were combined and freeze‐dried.

#### 2.11.3 Reversed‐phase high‐performance liquid chromatography (RP‐HPLC)

After collection of freeze‐dried fraction based on Sephadex G‐25, it was dissolved into diH_2_O at 600 mg/mL; later, the 20 μL system was applied to RP‐HPLC analysis using the C18 column (Zorbax 9.4 × 250 mm, Agilent Technologies, Shanghai). Then, the sample was eluted using solution A (including 0.1% trifluoroacetic acid TFA within diH_2_O) as well as solution B (including 0.1% TFA contained within acetonitrile) within 20 min at the 5%–30% B gradient and the 1.0 mL/min flow rate. Eluate OD values were detected at 215 nm. This purification procedure was repeated until sufficient samples were collected for further study.

### 2.12 Amino acid sequence analysis on peptides

The fraction that showed the greatest calcium‐chelating capacity obtained from RP‐HPLC was explored with the use of the Q Exactive Hybrid Quadrupole‐Orbitrap Mass Spectrometer (Thermo Fisher Scientific, USA). Those mass parameters were as follows: 2.2 kV spray voltage, 5 μL/min flow rate, and 300.0–1400.0 m/z mass range.

### 2.13 Peptides synthesis

The peptides were synthesized from China Peptides Co., Ltd (Shanghai, China) with purity higher than 98%. Thereafter, this work measured peptides' calcium‐binding ability.

### 2.14 Ultraviolet–visible (UV–vis) absorption spectroscopy

This work dissolved peptide and the peptide‐calcium chelate into diH_2_O separately at 1 mg/mL. Later, the UV–Vis spectrophotometer (UV‐3600plus, Shimadzu Co., Ltd., Japan) was utilized to record sample spectra over the wavelength of 200–400 nm. Prior to measurement, the UV–vis spectrophotometer was blank corrected with diH_2_O.

### 2.15 X‐ray diffraction (XRD) measurement

After even grinding the peptide and the peptide‐calcium chelate samples, they were added onto a sample plate and put into a holder. Later, the diffractometer (D8 advance, Brook AXS Co., Ltd., Germany) was utilized to collect diffraction data within the scanning angle range of 10°–80° at the 5 deg/min scanning speed.

### 2.16 Circular dichroism (CD)

Referring to the method of Lingyu Han (Han et al., 2024) with appropriate modifications using a Jasco J‐1500 CD spectrometer (JASCO, Japan), peptide and the peptide‐calcium chelate were dissolved in diH_2_O at a mass concentration of 1 mg/mL, respectively. Circular dichroism in a wavelength range (190–260 nm) was collected with an optical diameter of the colorimetric cell of 0.1 cm, a scanning rate of 200 nm/min, and diH_2_O as a blank correction, and measured three times to take the average value. CD spectra deconvolution software CDNN 2.1 software was used to examine the information contained in secondary structures.

### 2.17 Zeta potential analysis

The zeta potential of the peptide and the peptide‐calcium chelate was analyzed using a Zetasizer Nano ZS90 particle size analyzer (Malvern, UK). The measurement was performed as described previously in the method (Hu et al., 2022). Peptide and peptide‐calcium chelate (1 mg/mL) were added to a U‐shape cell and equilibrated for 60 s at 25°C.

### 2.18 Molecular dynamics (MD) simulation analysis

The peptides' original structures were obtained using the online peptide structure constructor PEP‐FOLD (https://bioserv.rpbs.univ‐paris‐diderot.fr/services/PEP‐FOLD/). Besides, MD simulation was conducted using Amber 14 and Amber 15 Tools (Gotz et al., 2012; Pierce et al., 2012; Salomon‐Ferrer et al., 2013). Both ions and peptides were presented using the forcefield “leaprc.ff14SB.” Thereafter, this system was put into the rectangular box that contained transferable intermolecular potential 3‐point (TIP3P) water (with the boundary being 10.0 Å) by adopting the “SolvateOct” command using the minimal inter‐solute atom distance. Subsequently, the peptide‐Ca^2+^ complex system was equilibrated with a GPU‐accelerated Particle Mesh Ewald Molecular Dynamics (PMEMD) module for brief minimization, under heating for 1000 ps and density equilibration for 500 ps using weak constraints. Finally, MD simulation for 100 ns was conducted at around 323 K (Wang et al., 2018).

### 2.19 Statistical analysis

All experiments were performed at least three independent replications, and the data were presented as the mean ± SD. The statistical analysis was performed using GraphPad Prism (Version 6.01, Graphad Inc., San Diego, California). Analysis of variance (ANOVA) was performed to determine the significance of the main effects. A value of *p* < .05 was considered statistically significant.

## 3 RESULTS AND DISCUSSION

### 3.1 Effects of different enzymes and enzymatic conditions on antler bone hydrolysis

**FIGURE 1:**
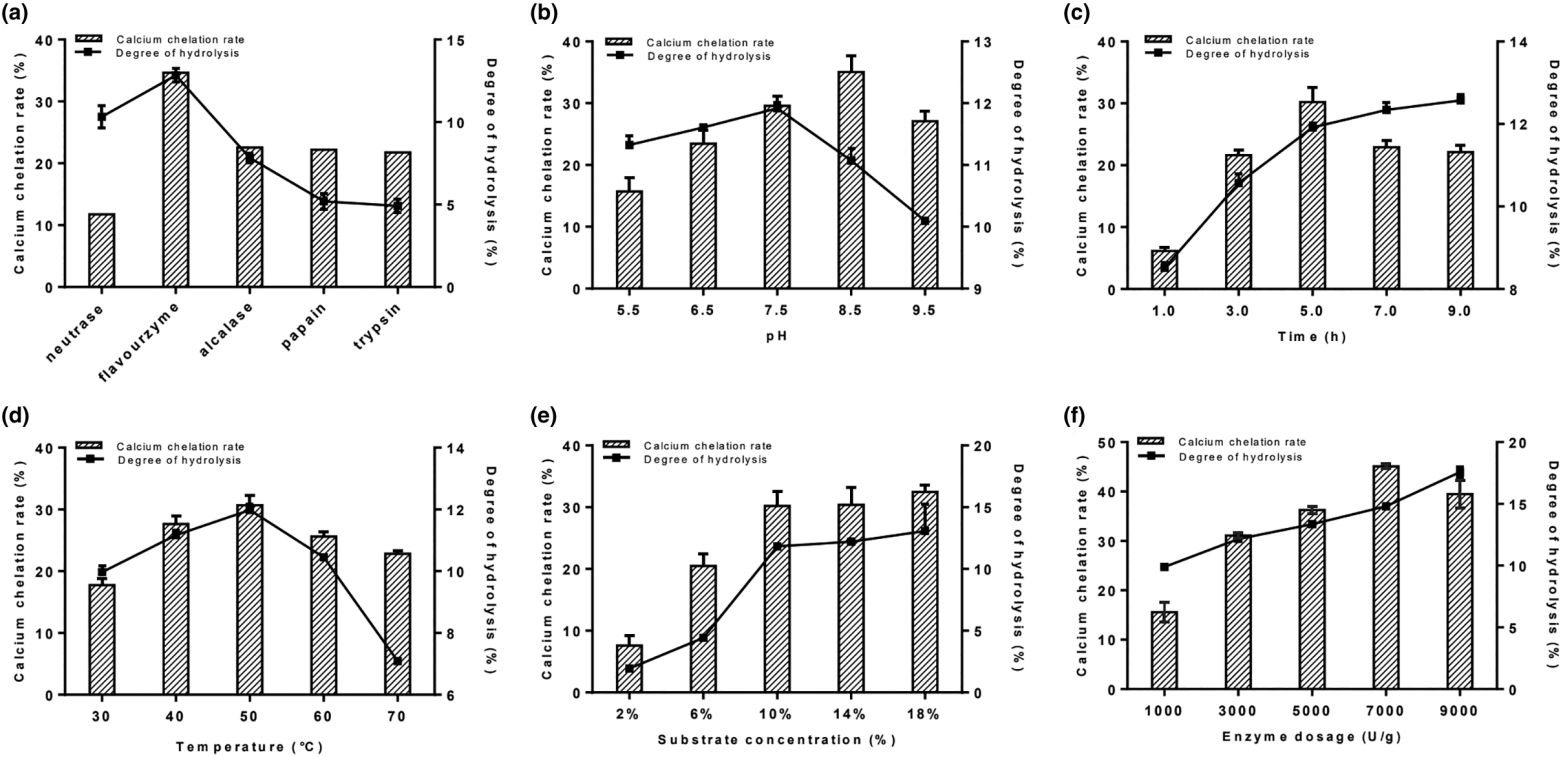
Effects of (a) different enzymes, (b) pH, (c) time, (d) temperature, (e) substrate concentration, and (f) enzyme dosage on calcium‐chelating capacity and degree of hydrolysis.

In the study, the antler bone was hydrolyzed by five different enzymes (neutrase, flavourzyme, alcalase, papain, and trypsin). As shown in Figure 1a, the calcium‐binding capacity of the hydrolysate prepared by flavourzyme reached 34.67 ± 1.24%, which was significantly higher than that of the hydrolysates prepared by the other four proteases (*p* < .01) and even reached approximately three times that of the hydrolysate prepared by neutrase. Coincidentally, the highest degree of hydrolysis was also the hydrolysate prerared by flavourzyme (12.83 ± 0.40%). The proper hydrolysis might facilitate the exposure of metal‐binding sites and thus contribute to the calcium combination (Chen et al., 2014; Lin et al., 2015). Therefore, flavourzyme was considered the most suitable enzyme for the preparation of calcium‐binding peptides. In addition, other studies found that the hydrolysates prepared by flavourzyme derived from porcine plasma and chlorella also showed a high calcium chelating rate (Jeon et al., 2010; Lee & Song, 2009).

**FIGURE 2:**
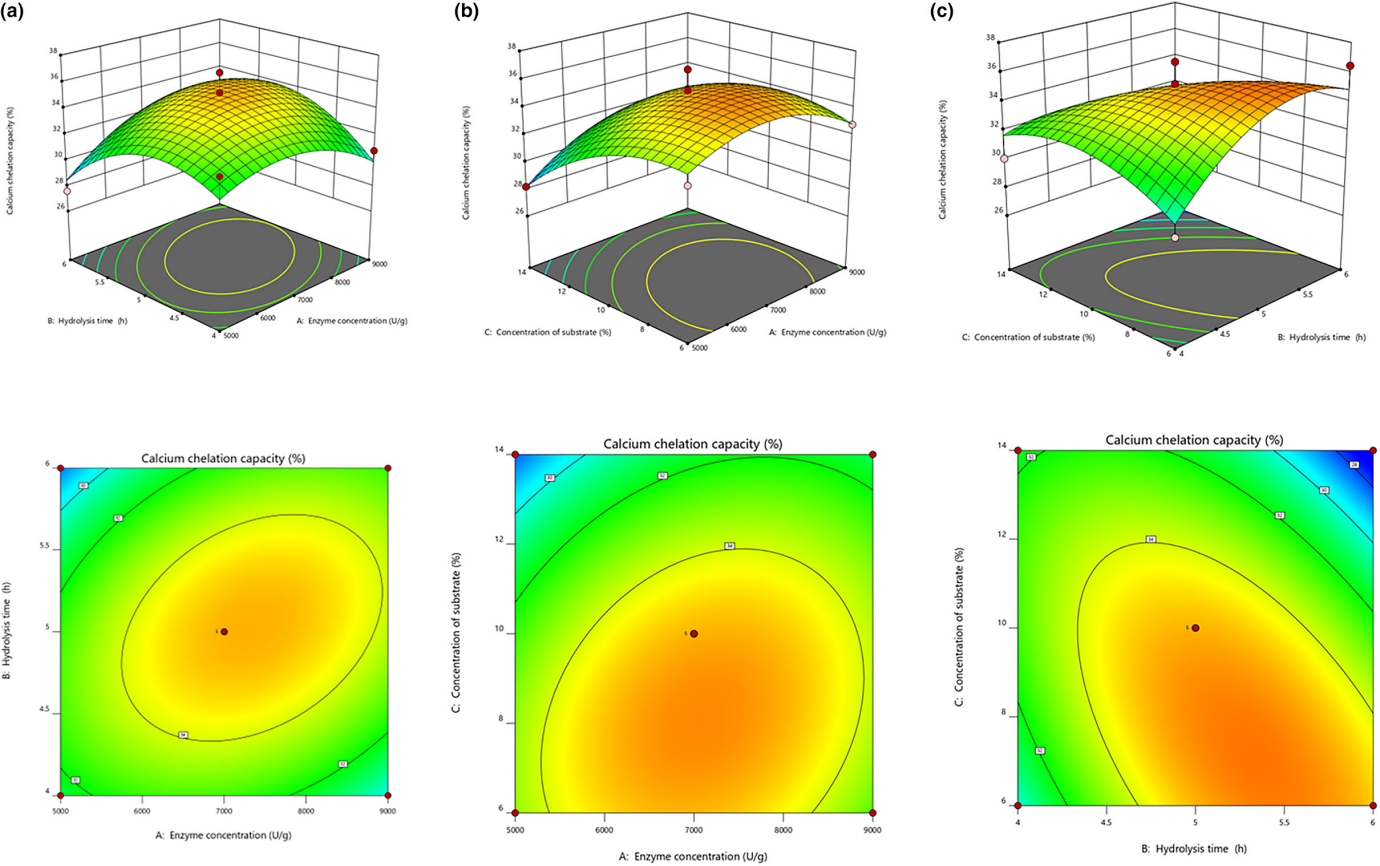
Response surface of calcium chelation rate. (a) Effects of enzyme concentration and hydrolysis time on calcium chelation rate, (b) effects of enzyme concentration and concentration of substrate on calcium chelation rate, and (c) effects of hydrolysis time and concentration of substrate on calcium chelation rate.

The single‐factor tests were used to determine the optimal enzymatic conditions for flavourzyme. The calcium chelating rate first increased and then reached equilibrium with an increase in substrate concentration (Figure 1e). In the other four‐factor ranges, the calcium chelating rate initially reached a maximum and then decreased. The degree of hydrolysis (DH) first increased and then decreased with an increase in pH and temperature (Figure 1b,d). It was observed that the DH increased with the increase in other three single factors. As shown in Figure 2c, the DH increased with the prolongation of the enzymatic hydrolysis time, and the rate of increase was first fast and then slow, which was due to the continuation of the enzymatic reaction, and the hydrolysis product would have a feedback inhibition effect on the reaction when it reached the peak (Gerhart & Pardee, 1962), which pushed the reaction dynamic equilibrium and made the degree of hydrolysis tend to be stabilized (Zhong et al., 2023). Calcium chelating rate showed an increasing and then decreasing trend with the increase of enzymatic hydrolysis time, reaching a peak at 5 h. Calcium chelating rate continued to show a decreasing trend with the enzymatic hydrolysis time. DH increased with increasing enzyme dosage. Calcium chelating rate increased and then decreased with increasing enzyme dosage (Figure 1f). The following conditions were determined to be ideal for hydrolysis: pH of 8.5, hydrolysis time of 5.0 h, temperature of 50°C, substrate concentration of 10%, and enzyme dosage of 7000 U/g.

### 3.2 Response surface methodology (RSM) to optimize calcium chelation capacity

Taking the calcium chelation capacity as the dependent variable *Y*, three factors, namely, enzyme concentration, hydrolysis time, and concentration of substrate, were selected from the results of the single‐factor test to carry out the BBD and RSM experimental designs, and the results are shown in Table 1. A second‐order polynomial equation was employed to forecast the calcium chelation capacity (%), considering the impact of these three variables. The function is given by:

$$\begin{array}{cc} Y\;\left(\%\right)=\; & 35.05+0.6175A–0.1125B–1.70C+1.39\mathrm{AB}\\ & +0.9050\;\mathrm{AC}–2.48\;\mathrm{BC}–1.92A^{2}–2.54B^{2}–1.72C^{2} \end{array}$$

where *Y* is the calcium chelation capacity, *A* is the enzyme concentration, *B* is the hydrolysis time, and *C* is the concentration of substrate.

As shown in Table 2, the regression model was examined by ANOVA using *F*‐test and *p*‐test. The model *p*‐value (0.0228, *p* < .05) showed that the model provides statistically significant results. Thus, the model is suitable. The values of *R* ^2^, *R*_adj_^2^, and lack of fit were 0.8652, 0.6919, and 0.1180 (*p* > .05), which showed that the experimental error was minimized and the model fitted well. The optimal enzymatic hydrolysis process of BC was determined based on the results of regression modeling. The theoretical optimal enzymatic process conditions were: enzyme concentration of 7040.06 U/g, substrate concentration of 11.3849%, enzymatic time of 4.79556 h, and calcium chelation capacity of 34.3582% for enzymatic products (Figure 2). Then the enzymatic process was adjusted with enzyme dosage of 7000 U/g, reaction temperature of 50°C, pH of 8.5, substrate concentration of 10%, and enzymatic time of 4 h. In order to validate the process, three replications were carried out, and the final calcium chelation capacity was 34.31 ± 0.27%, which was close to the theoretical value.

**TABLE 2:** Analysis of variance of response surface regression model.

Variables	Sum of squares	df	Mean square	*F*‐value	*p*‐Value
Model	123.29	9	13.70	4.99	.0228^a^
A	3.05	1	3.05	1.11	.3267
B	0.1013	1	0.1013	0.0369	.8531
C	23.05	1	23.05	8.40	.0230^a^
AB	7.78	1	7.78	2.84	.1360
AC	3.28	1	3.28	1.19	.3107
BC	24.50	1	24.50	8.93	.0203^a^
A^2^	15.59	1	15.59	5.68	.0486^a^
B^2^	27.25	1	27.25	9.93	.0161^a^
C^2^	12.44	1	12.44	4.53	.0707
Residual	19.21	7	2.74		
Lack of fit	14.15	3	4.72	3.73	.1180
Pure error	5.06	4	1.26		
Cor total	142.50	16			
*R* ^2^ = 0.8652			*R*_adj_^2^ = 0.6919		

^a^ Significant within a 95% confidence interval.

### 3.3 Structural characterization

**FIGURE 3:**
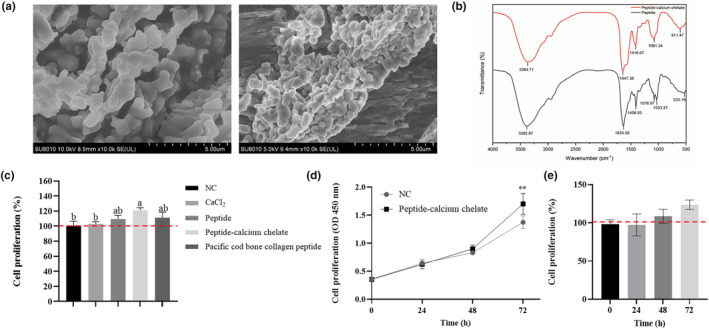
Structural characterization of peptide‐calcium chelate and its effect on the proliferation rate of MC3T3‐E1 cells. (a) SEM image of peptide (×10,000 magnification) and peptide‐calcium chelate (×10,000 magnification); (b) FTIR spectra of peptide (black line) and peptide‐calcium chelate (red line) over the wave number range from 4000 to 400 cm^−1^; (c) effects of different treatment groups on the proliferation of MC3T3‐E1 cells; (d and e) effect of peptide‐calcium chelate on the proliferation rate of MC3T3‐E1 cells. The values are shown as the means ± SDs, *n* = 3. Different letters (a, b) indicate a significant difference (*p* < .05). Values significant in comparison with control with **p < .01.

From Figure 3a, peptide presented a loose and porous structure; after chelating with calcium, peptide‐calcium chelate exhibited clusters. It may be the calcium adsorbed on the peptide. Changes of FTIR absorption peaks could reveal that calcium ions interacted with ligand groups within peptides, and this was significant for investigating calcium‐chelating peptide generation and constitution (Zhao, Huang, Cai, et al., 2014). In Figure 3b, differences in FTIR spectra of peptide and peptide‐calcium chelate were of significance. The high frequency absorption at 3382.87 cm^−1^ in peptide was associated with N‐H stretching vibration, and the wavenumber shifted to 3364.71 cm^−1^ following chelation with calcium ions, indicating the more potent N‐H's electron cloud density because of dipole field or inductive effect (El Hajjouji et al., 2007). The red shift implied the increasing of N‐H stretching frequencies and revealed that N‐H group was involved in the complicated generation (Moritz, 1962). The amide I bands were 1635.55 cm^−1^, which related to the stretching vibration of the carbonyl group (C=O). The bands shifted to 1647.38 cm^−1^ after binding with calcium ions. It suggested that the carboxylic group of the peptide was related to peptide‐calcium chelate generation. Additionally, the peak at 1408.53 cm^−1^ shifted to 1081.34 cm^−1^ when the peptide chelated with calcium ions, which was associated with C‐O‐Ca formation (Lin et al., 2015). These results demonstrated that peptides bind to calcium mainly through amino nitrogens atom and carboxyl oxygen atoms, consistent with calcium‐binding peptides collected in whey protein hydrolysate (Zhao et al., 2014a).

### 3.4 Effects of peptide‐calcium chelate on MC3T3‐E1 cells

**FIGURE 4:**
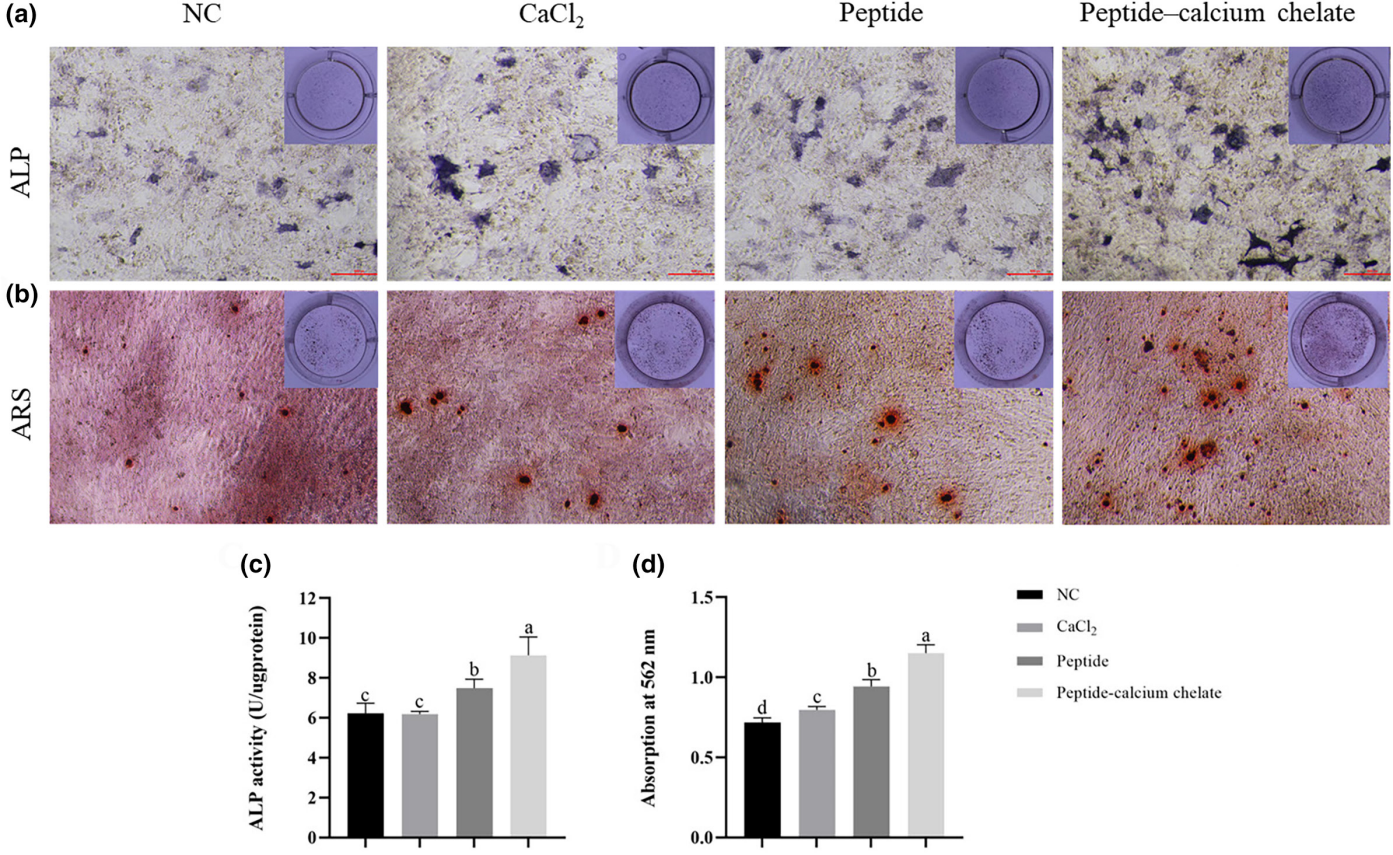
Effect of peptide‐calcium chelate on differentiation and mineralization of MC3T3‐E1 cells. (a and b) Representative images of ALP and ARS staining of MC3T3‐E1 cells; (c) quantitative analysis of the ALP activity in MC3T3‐E1 cells; (d) quantitative analysis of calcium mineralization in MC3T3‐E1 cells. The values are shown as the means ± SDs, *n* = 3. Different letters (a–d) indicate a significant difference (*p* < .05).

Proliferation of MC3T3‐E1 cells is an important part of their osteogenic activity and is the basis for subsequent differentiation and mineralization of MC3T3‐E1 cells. The proliferation of peptide‐calcium chelate for MC3T3‐E1 cells was evaluated by the CCK‐8 assay. As shown in Figure 3c, compared with the NC group, the proliferative bioactivity of peptide‐calcium chelate significantly increased (*p* < .05). Compared with the NC group, CaCl_2_, peptide, and pacific cod bone collagen peptide did not show a significant difference, respectively. In addition, the effects of peptide‐calcium chelate on the proliferation of MC3T3‐E1 cells are shown in Figure 3d,e. Compared with the NC group, peptide‐calcium chelate significantly increased the proliferation of MC3T3‐E1 cells at 72 h (*p* < .01). Previous studies have found similar results in chelates from cattle bone, porcine bone, and tilapia bone, where the peptide calcium chelates promoted MC3T3‐E1 cell proliferation through activation of the PI3K/Akt pathway and significantly facilitated calcium uptake. ALP activity is a common marker of the early stages of osteoblast differentiation, whereas the formation of bone mineralization nodules is one of the most important phenomena in the final stages of osteoblast differentiation and represents the maturation of osteoblasts. As shown in Figure 4a, peptide‐calcium chelates significantly stimulated ALP activity in MC3T3‐E1 cells after 7 days of osteogenic induction and significantly increased the formation of mineralized nodules after 28 days (Figure 4b). Quantitative analyses of ALP activity and calcium deposition confirmed these findings (Figure 4c,d). This result is consistent with the findings of Shen et al. (2021) that peptide‐calcium chelate may promote ALP expression through the AMPK, the PI3K‐Akt, the MAPK, and Wnt signaling pathways (Huang et al., 2022). Peptide‐calcium chelate may contribute to the mineralization of MC3T3‐E1 cells by promoting the expression of *OGN*, *APOD*, *IGF‐1*, *OPN*, *OCN*, *RUNX2*, *OSX*, and *COL‐I* (A et al.; Huang et al., 2022). Peptide‐calcium chelate may activate calcium‐sensing receptor, increase intracellular calcium concentration, and activate TGF‐β1/Smad2/3 signaling pathway (Qi et al., 2024).

### 3.5 Isolation and purification of peptides from antler bone hydrolysate

**FIGURE 5:**
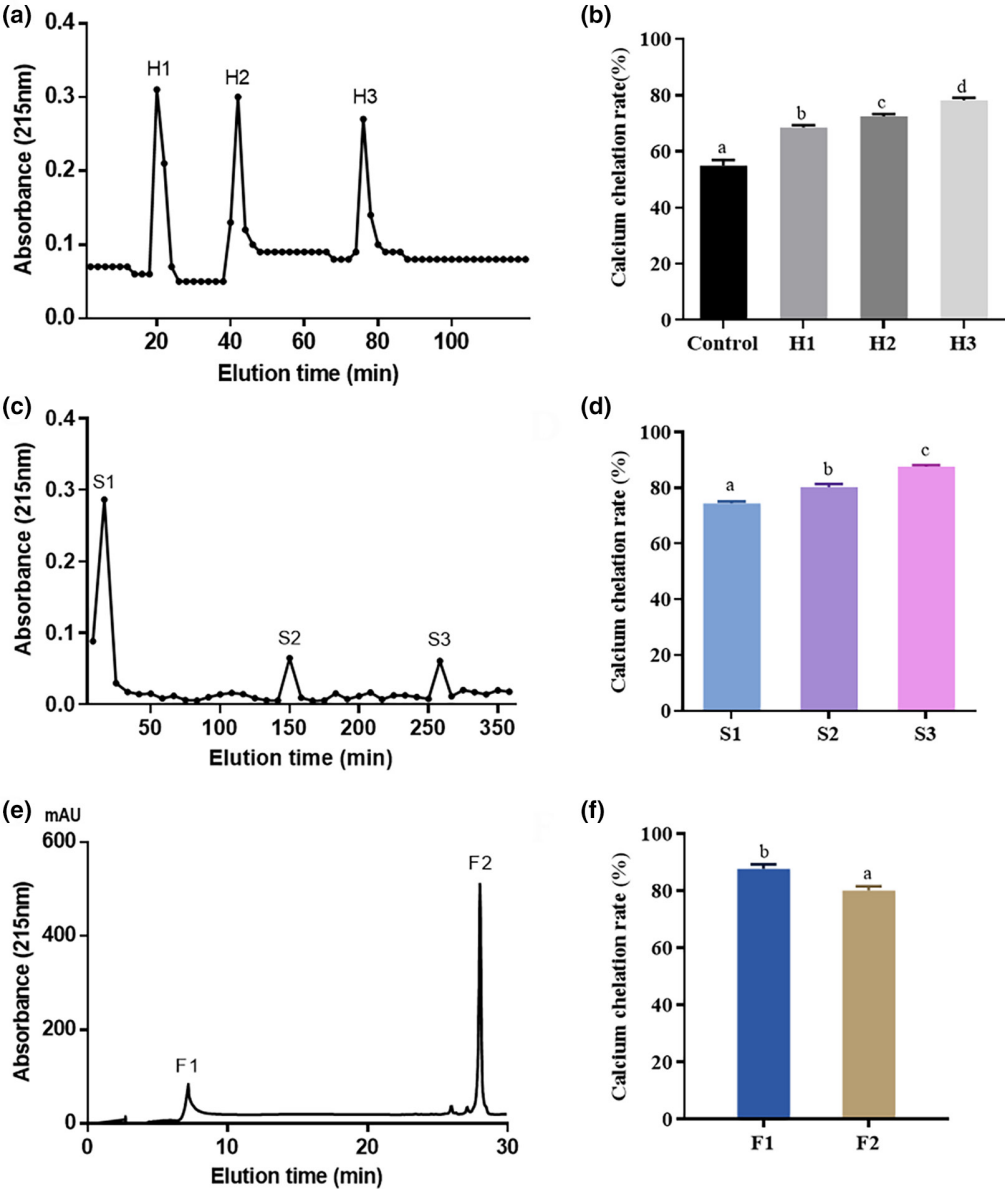
The HAC profile of antler bone hydrolysate and the calcium chelation rate. (a) Fractions (H1, H2, and H3) separated from antler bone hydrolysate. (b) Calcium chelation rate of control (antler bone hydrolysate), H1, H2, and H3. The gel filtration chromatography profile of H3 and the calcium chelation rate. (c) Fractions (S1, S2, and S3) separated from H3. (d) Calcium chelation rate of S1, S2, and S3. The RP‐HPLC profile of S3 and the calcium chelation rate. (e) Fractions (F1 and F2) separated from S3. (f) Calcium chelation rate of F1 and F2. The values are shown as the means ± SDs, *n* = 3. Different letters (a–d) indicate a significant difference (*p* < .05).

To isolate peptide‐calcium chelate, the hydrolysate was purified using a hydroxyapatite chromatography column (Hilbrig & Freitag, 2012; Hou et al., 2018). Antler bone peptides with high calcium chelating rates exhibited adhesion to calcium ions on the surface of hydroxyapatite crystals. As shown in Figure 5a, three fractions (H1, H2, and H3) were obtained, and the calcium chelating capacity of the H3 fraction was up to 78.16 ± 0.93% (Figure 5b), which was higher than deer bone calcium‐chelating peptide (Bi et al., 2018). This fraction was collected for further purification.

Molecular weight of the peptide could influence the binding ability of calcium ions (Chaud et al., 2002). The current study shown that peptides with molecular weights below 2 kDa have a high calcium binding capacity (Choi et al., 2012; Lv et al., 2008; Wang et al., 2018). The H3 fraction was separated according to its molecular weight by Sephadex G‐25 column. The three different peaks were obtained, named S1, S2, and S3, respectively (Figure 5c). Fraction S3 showed the highest calcium chelating capacity (Figure 5d), and it was collected for subsequent purification.

The S3 fraction was further separated by an RP‐HPLC system equipped with a C‐18 column, using a linear gradient from 5% to 30% acetonitrile containing 0.1% TFA at a flow rate of 2.0 mL/min, depending on different polarities. Subsequently, two major fractions with high response values, named F1 and F2, were observed (Figure 5e). The fraction F1 exhibited a high calcium chelating capacity of 87.63 ± 1.64% (Figure 5f), which was higher than that of the peptide from wheat germ protein hydrolysates (84.45 ± 1.49%) and collagen peptide from pig bone (78.01 ± 0.24%) (Wang et al., 2018; Wu et al., 2019b).

### 3.6 Identification of the calcium‐binding peptides

**FIGURE 6:**
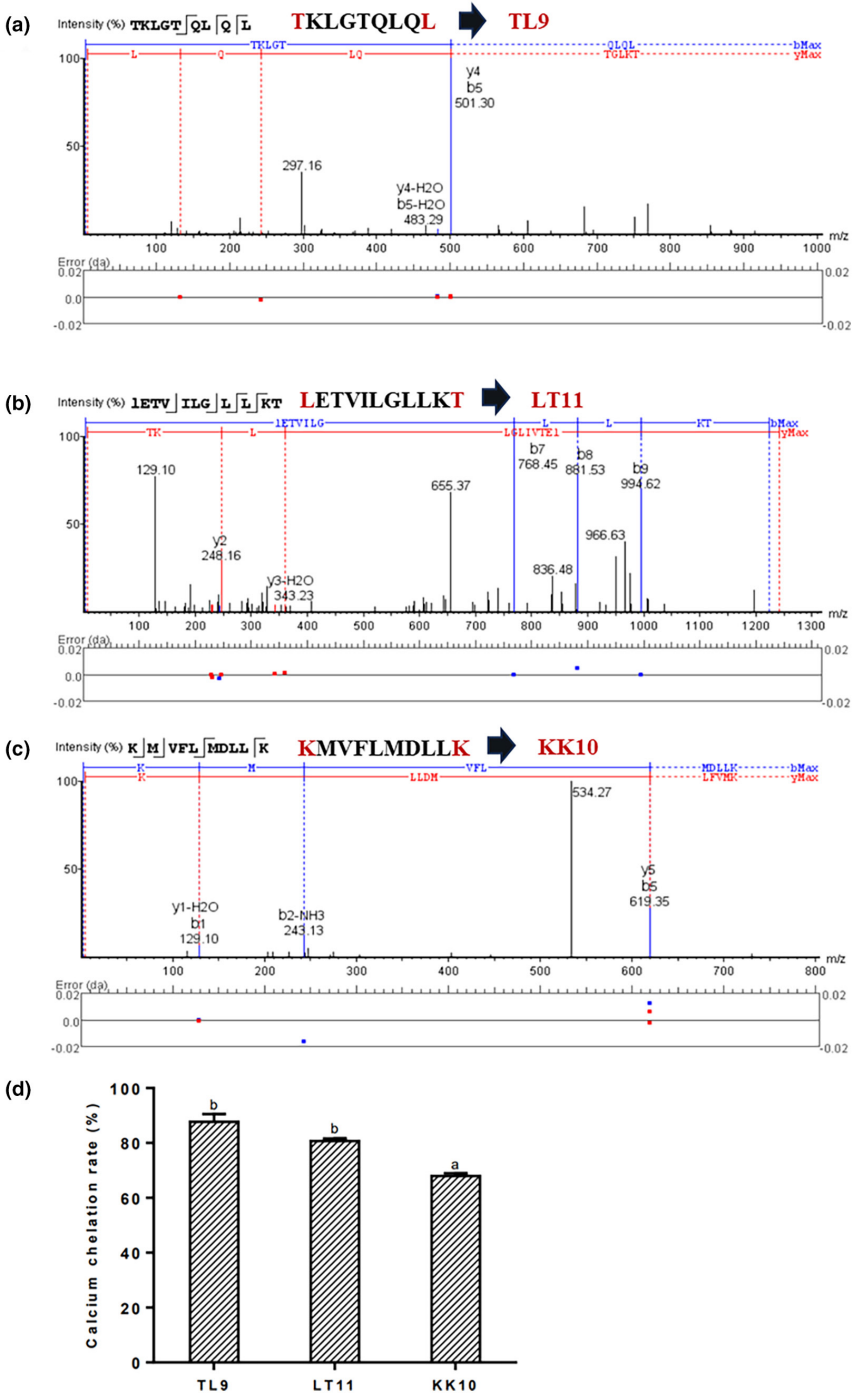
Peptides from fraction F1 identified using LC–MS/MS. MS/MS spectra of the peptide (a) TL9, (b) LT11, and (c) KK10. (d) Calcium chelation rate of commercially synthesized peptides TL9, LT11, and KK10. The values are shown as the means ± SDs, *n* = 3. Different letters (a, b) indicate a significant difference (*p* < .05).

Fraction F1 obtained from RP‐HPLC was sequenced for its amino acid by LC–MS/MS. The sequences of the calcium‐chelating peptides were identified as TKLGTQLQL, LETVILGLLKT, and KMVFLMDLLK, which were named as TL9, LT11, and KK10, respectively (Figure 6). The sequences correspond well to the Uniprot database, and the complete description is detailed in Table 3. According to current reports, these three peptides were the first reported novel calcium‐chelating peptides derived from *Cervus elaphus*. The three peptides were subsequently synthesized with purity higher than 98% from ChinaPeptides Co., Ltd. The calcium chelating rate of TL9, LT11, and KK10 reached 87.68 ± 2.86%, 80.72 ± 0.93%, and 67.97 ± 0.98% (Figure 6d). The main factors that affect the calcium‐binding capacity of peptides were the composition and sequence of amino acids (Budseekoad et al., 2018; Charoenphun et al., 2013). One of the two peptides with high calcium chelating rate, TL9, presenced Thr(T) at the C‐terminal and Leu(L) at the N‐terminal, while the other one, LT11, presenced Leu(L) at the C‐terminal and Thr(T) at the N‐terminal. It has been reported that the hydrophilic amino acids (T, K, and E) and hydrophobic amino acids (L, I, and G) on the peptide chain play important roles in calcium‐binding (Liu et al., 2013; Liu et al., 2016; Malison et al., 2021). Thr(T) was identified as a functional binding site of wheat germ protein peptide to calcium (Wang et al., 2018). Egg white peptide containing Thr(T) has been shown to promote calcium absorption (Sun et al., 2017). In addition, studies have suggested that the hydrophobic amino acid Leu(L) facilitated calcium binding (Liu et al., 2013). For example, the amino acid sequence of the calcium binding peptide from defatted *Schizochytrium* sp. protein hydrolysates was determined to be YL (Cai et al., 2017). Interestingly, Siriporn Budseekoad et al. (2018) found that Leu(L) appearing at the C‐terminal, N‐terminals, or within the peptide chain led to better calcium‐binding capacity when compared to peptides that contained Leu(L) at both the C‐ and N‐terminals, which was consistent with the results of this study. Therefore, Thr(T), Leu(L), and their positions on the peptide chain might be the main factors for the calcium‐chelating properties of the peptides identified in this study.

**TABLE 3:** Description of the Identified Peptides from UniProt.

Sequence	m/z	Organism	Protein
TKLGTQLQL	501.3046	*Cervus elaphus*	Toll‐like receptor 3 short‐type
LEVTILGLLKT	621.3897	*Cervus elaphus*	Annexin A2
KMVFLMDLLK	619.3576	*Cervus elaphus*	Excision repair cross‐complementing rodent repair deficiency complementation group 6‐like protein

### 3.7 FTIR spectroscopy of peptide and peptide‐calcium chelate

**FIGURE 7:**
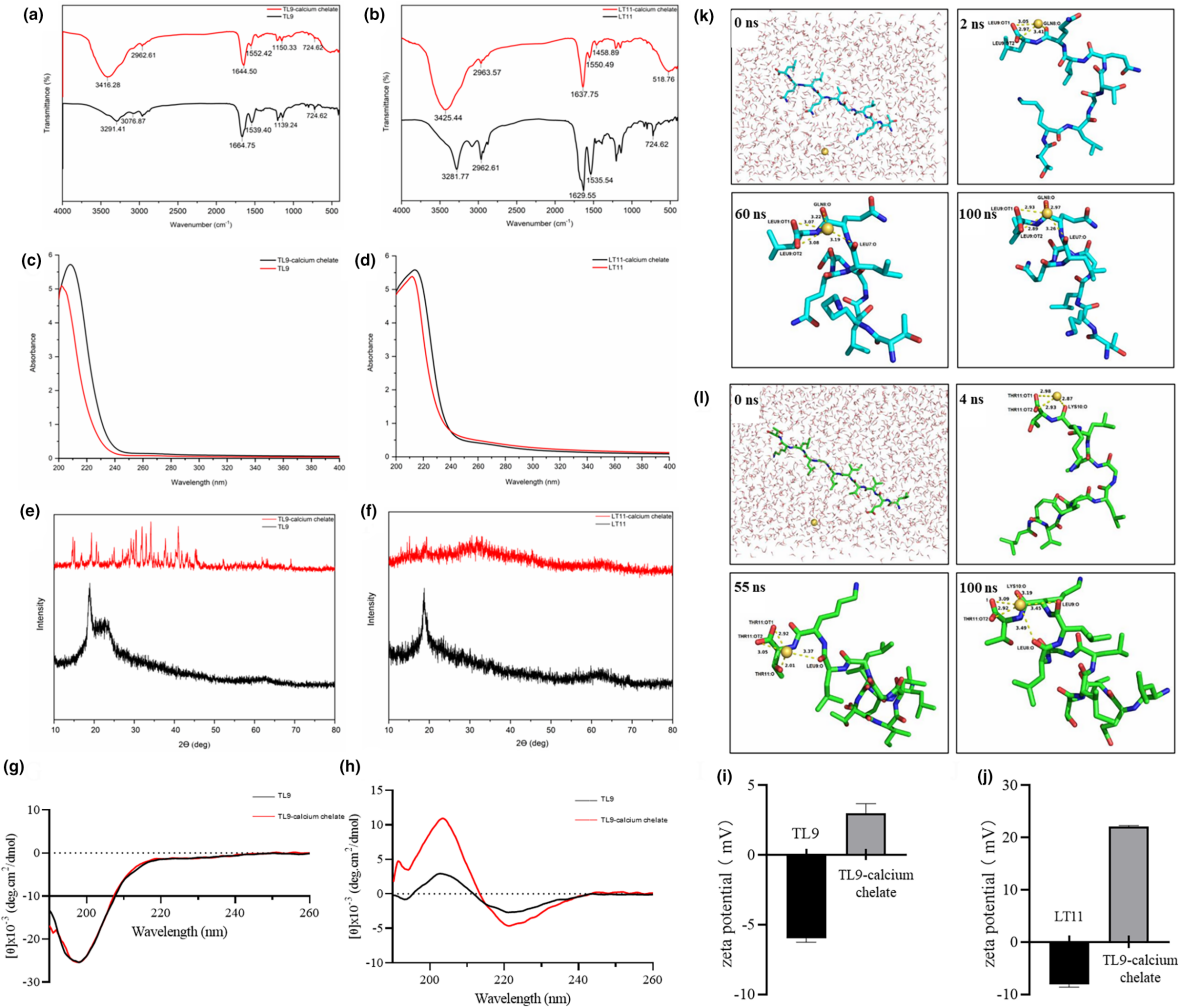
(a and b) FTIR spectra of TL9 and LT11 and their calcium chelate in the regions from 4000 to 400 cm^−1^. (c and d) UV–Vis spectra of TL9 and LT11 and their calcium chelate in the regions from 200 to 400 nm. (e and f) XRD spectra of TL9 and LT11 and their calcium chelate in the regions of 10–80 °. (g and h) CD spectroscopy of TL9 and LT11 and their calcium chelate complex over the range of 190 nm to 260 nm. (i and j) Zeta potential profile of the TL9 and LT11 and their calcium chelate by a Zetasizer Nano ZS90 particle size analyzer. (k) Snapshots of molecular dynamic simulation of TL9‐calcium chelate at 0 ns, 2 ns, 60 ns, and 100 ns. Color code: Yellow‐orange, calcium; red, oxygen; blue, nitrogen; cyan, carbon. In order to exhibit the clear conformation of peptide‐calcium chelate, water was not shown in 2 ns, 60 ns, and 100 ns snapshots. (l) Snapshots of molecular dynamic simulation of LT11‐calcium chelate at 0 ns, 4 ns, 55 ns, and 100 ns. Color code: Yellow‐orange, calcium; red, oxygen; blue, nitrogen; green, carbon. In order to exhibit the clear conformation of peptide‐calcium chelate, water was not shown in 4 ns, 55 ns, and 100 ns snapshots.

In Figure 7a,b, differences in FTIR spectra of peptide and peptide‐calcium chelate were of significance. The high frequency absorption at 3291.41 cm^−1^ in TL9 and 3281.77 cm^−1^ in LT11 was associated with N‐H stretching vibration, and wavenumber shifted to 3416.28 cm^−1^ and 3425.44 cm^−1^ following chelation with calcium ions, indicating the more potent N‐H's electron cloud density because of dipole field or inductive effect (El Hajjouji et al., 2007). The amide I bands were 1664.75 cm^−1^ (TL9) and 1629.55 cm^−1^ (LT11), which related to the stretching vibration of the carbonyl group (C=O). The bands shifted to 1644.50 cm^−1^ (TL9‐calcium chelate) and 1637.75 cm^−1^ (LT11‐calcium chelate) after binding with calcium ions. It suggested that the carboxylic group of TL9 and LT11 was related to peptide‐calcium chelate generation. Two weak absorption peaks detected at 1539.40 cm^−1^ in TL9 and 1535.54 cm^−1^ in LT11, representing the amide II band coupled by N‐H binding vibrations and C‐N stretching vibrations, red‐shifted to the higher frequency region at 1552.42 cm^−1^ (TL9‐calcium chelate) and 1550.49 cm^−1^ (LT11‐calcium chelate). Additionally, the peak at 1139.24 cm^−1^ shifted to 1150.33 cm^−1^ when TL9 chelated with calcium ions. In the fingerprint region of LT11, the peak at 724.62 cm^−1^ shifted to 518.76 cm^−1^, which resulted from N‐H and C‐H bonds' stretching vibrations, indicating that N and O atoms form coordination bonds with calcium ions (Wu et al., 2017). These results demonstrated that both TL9 and LT11 bind to calcium mainly through amino nitrogen atoms and carboxyl oxygen atoms.

### 3.8 UV–Vis absorption spectroscope of peptide and peptide‐calcium chelate

The substance structure could be analyzed by monitoring those intensity alterations and shifts in UV bands. As shown in Figure 6c,d, the UV spectra of TL9 and LT11 showed obvious differences compared with those of their calcium chelate. The maximum absorptions exhibited at 202 nm in TL9 (Figure 7c) and 212 nm in LT11 (Figure 7d) were deemed to be the amide bond's characteristic peaks and C=O's *n* → π* transition within the peptide bond, respectively (Yu & Fan, 2012). Following calcium chelation, absorption peak intensity notably increased, with obvious red shifts on the spectrum of TL9‐calcium cheate and LT11‐calcium chelate. The results of UV spectroscopy demonstrated that auxochromes (–OH and –NH_2_) and chromospheres (C=O and –COOH) of peptides changed after combining with the calcium ions (Armas et al., 2006). These UV spectra phenomena were in accordance with previous studies (Zhao et al., 2014a). Based on the above experimental results, TL9 and LT11 are bound to calcium ions mainly through the nitrogen of the amino group and the oxygen atom of the carboxyl group.

### 3.9 XRD spectra of peptide and peptide‐calcium chelate

XRD was an analytical method for investigating the structure of substrance by crystalline alterations (Chander et al., 2020). XRD spectra observed from Figure 7e,f showed the different characteristics of crystals in peptides and their calcium chelate. From TL9's spectrum, one strong and broad peak in this band occurred between 2Ɵ 18° and 24° (Figure 7e), indicating the random amorphous structure of TL9. After binding with calcium, many new sharp peaks appeared and the existing broad peak broke into 6 small peaks. Such alterations suggested that the novel crystalline architecture was formed (Malison et al., 2021). In the spectrum of LT11, one sharp peak appeared at approximately 2Ɵ 20° (Figure 7F), which showed an amorphous structure of LT11. Compared with LT11, the sharp characteristic peak in LT11‐calcium chelate disappeared and was substituted by many small diffusion peaks, which implied that the increase in scattering intensity might be associated with junctional zone formation and chain associations. These changes on the XRD spectrum, including the disappearance/break of existing peaks or the appearance of novel absorption peaks, revealed that complexes of organic ligands with calcium ions were formed (Malison et al., 2021; Wang et al., 2018).

### 3.10 CD spectral analysis of peptide and peptide‐calcium chelate

CD is currently the most widely used method for determining the secondary structure of proteins (Beyer et al., 2004). Conformational changes in the secondary structure of a protein or peptide are accompanied by changes in cyclic secondary spectral position and absorption (Greenfield, 2006). The secondary structures of peptide and their calcium chelate were analyzed by CD, as shown in Figure 7g and Table 4. The absorption peaks of the peptides were not shifted after the formation of the peptide‐calcium complexes; the mass fractions of α‐helix, β‐sheet, β‐turn, and randon‐coil of TL9 were 5.7%, 71.9%, 15.5%, and 6.8%, respectively; after binding with calcium, the content accounted of α‐helix (5.1%), β‐sheet (71.2%), β‐turn (16.9%), and randon‐coil (6.8%) did not change significantly, suggesting that the secondary structure of the peptide‐calcium chelates was not changed during the formation process. As shown in Figure 7h and Table 4, after the formation of the peptide‐calcium complexes, the absorption peaks of the peptides were shifted, and the content accounted of α‐helix, β‐sheet, β‐turn, and randon‐coil of LT11 were 0%, 55.4%, 15.5%, and 6.8%, respectively; after binding with calcium, the α‐helix content did not change significantly, but the β‐sheet increased to 94.8%, β‐turn content accounted decreased to 5.2%, and randon‐coil content accounted decreased to 0, which indicated that Ca^2+^ induces changes in the spatial structure of peptides. This phenomenon is consistent with the observation of Kai Zhang (Zhang et al., 2019) that the loose secondary structure of cod peptide became a more compact secondary structure after chelating with Ca^2+^.

**TABLE 4:** Type of secondary structure relative content of α‐helix, β‐sheet, β‐turn, and random‐coil.

Name	Secondary structure (%)			
	α‐Helix	β‐Sheet	β‐Turn	Randon‐coil
TL9	5.7	71.9	15.5	6.8
TL9‐calcium chelate	5.1	71.2	16.9	6.8
LT11	0	54.4	11.6	33.9
LT11‐calcium chelate	0	94.8	5.2	0

### 3.11 Zeta potential analysis of peptide and peptide‐calcium chelate

Zeta potential measurement is crucial for assessing the surface charge properties of particles or molecules (Luo et al., 2020). Ca^2+^ binds primarily to free amino and carboxyl groups in peptides, and in the process of binding may lead to changes in the nature of the charge on the surface of the peptide partial ionization of amino acid residues produces a charge on the surface of the protein (Sun et al., 2020). As shown in Figure 7i, the zeta potential of TL9‐calcium chelate decreased significantly from 2.98 ± 0.71 to −5.97 ± 0.29 mV (*p* < .05) compared to TL9. As shown in Figure 7j, the zeta potential of LT11‐calcium chelate decreased significantly from 22.13 ± 0.15 to −8.06 ± 0.58 mV (*p* < .05) compared to LT11. These data suggest that both TL9 and LT11 undergo electron transfer when reacting with Ca^2+^, respectively, to produce new compounds.

### 3.12 MD simulation analysis of peptide and peptide‐calcium chelate

MD was conducted to explore dynamic changes in system structure. Peptide's original architecture was acquired through PEPFOLD, then molecular dynamic simulation was carried out using Amber14. Side‐chain orientation and alterations were essential during metal binding. Figure 7k displays snapshots (0, 2, 60, and 100 ns) regarding MD simulations for TL9‐calcium chelate. As a result, calcium ion's chelating site included carbonyl in Leu‐7, Gln‐8, and Leu‐9 residues. Noteworthily, the TL9 conformation is always changing, leading to the constantly changing distance between Leu‐7/Gln‐8 residues and calcium ions. Nonetheless, the distance between carboxyl oxygen in Leu‐9 residue and calcium ion did not show significant alteration within 2–100 ns. The bond strength of calcium‐oxygen (Leu‐9) was within 2.89–3.08 Å. These results of molecular dynamics were similar to those of mass spectrometry.

Figure 7l displays snapshots (0, 4, 55, and 100 ns) showing MD simulations for LT11‐calcium chelate. As a result, calcium ion's chelating site included carbonyl in the Leu‐8, Leu‐9, Lys‐10, and Thr‐11 residues. The LT11's conformation showed constant change; accordingly, the distance between Leu‐8/Leu‐9/Lys‐10 residues and calcium ions also exhibited constant changes. In snapshots at 4 ns, the carbonyl “O” of Lys‐10 residue chelated with calcium ions, while the chelation disappeared at 55 ns. The chelation was formed again at 100 ns. The bond length of calcium‐oxygen (Thr‐11) was within 2.92–3.09 Å, suggesting that the distance between the calcium ion and carboxyl oxygen of the Thr‐11 residue remained unchanged within 4–100 ns. In this study, the calcium ion might be more likely to generate complexes together with carboxylate groups of Leu or Thr with negative charge relative to amino nitrogen atoms, thus providing useful evidence for future research on the chelation of peptides with calcium ion.

## 4 CONCLUSION

In conclusion, peptide‐calcium chelate could significantly promote the proliferation, differentiation, and mineralization of MC3T3‐E1 cells. This work separated 3 calcium‐chelating peptides (TL9, LT11, and KK10) in the antler bone hydrolysate, among which TL9 and LT11 were verified to have high calcium chelating rates of 87.68 ± 2.86% and 80.72 ± 0.93%, respectively. Amino group nitrogen atom and carboxyl oxygen atom were related to chelating reaction, as suggested by FTIR, UV–vis, XRD, CD, and zeta potential. In addition, negatively charged carboxylate groups of Leu or Thr offered the main binding sites using MD simulation. Therefore, antler bone hydrolysate was a prospective source for the production of calcium‐binding peptides, which can improve bioactivity on MC3T3‐E1 cells.

## AUTHOR CONTRIBUTIONS

**Zhaoguo Wang:** Methodology (equal); software (equal); writing – original draft (equal). **Xiaorui Zhai:** Conceptualization (equal); resources (equal); writing – original draft (equal). **Xingyu Xiao:** Methodology (equal); software (equal). **Peijun Xia:** Methodology (equal); software (equal). **Xi Chen:** Validation (equal). **Yi Li:** Data curation (equal); resources (equal). **Linlin Hao:** Project administration (equal). **Yining Zhang:** Investigation (equal); project administration (equal).

## FUNDING INFORMATION

This work was supported by the Science and Technology Development Project of Jilin Province (20220204084YY).

## CONFLICT OF INTEREST STATEMENT

The authors declare that they have no known competing financial interests or personal relationships that could have appeared to influence the work reported in this paper.

## ETHICS STATEMENT

This study does not involve any human or animal testing.

## INFORMED CONSENT

Written informed consent was obtained from all study participants.

## Data Availability

Data will be made available on request.
